# Design of a Scalable and Fast YOLO for Edge-Computing Devices

**DOI:** 10.3390/s20236779

**Published:** 2020-11-27

**Authors:** Byung-Gil Han, Joon-Goo Lee, Kil-Taek Lim, Doo-Hyun Choi

**Affiliations:** 1 Electronics and Telecommunications Research Institute, Daegu 42994, Korea; kilyhan@etri.re.kr (B.-G.H.); leejg01679@etri.re.kr (J.-G.L.); ktl@etri.re.kr (K.-T.L.); 2 School of Electronics Engineering, Graduate School of Electronic and Electrical Engineering, Kyungpook National University, Daegu 41566, Korea

**Keywords:** edge-computing, light-weight, mobile device, object detector, scalable, SF-YOLO

## Abstract

With the increase in research cases of the application of a convolutional neural network (CNN)-based object detection technology, studies on the light-weight CNN models that can be performed in real time on the edge-computing devices are also increasing. This paper proposed scalable convolutional blocks that can be easily designed CNN networks of You Only Look Once (YOLO) detector which have the balanced processing speed and accuracy of the target edge-computing devices considering different performances by exchanging the proposed blocks simply. The maximum number of kernels of the convolutional layer was determined through simple but intuitive speed comparison tests for three edge-computing devices to be considered. The scalable convolutional blocks were designed in consideration of the limited maximum number of kernels to detect objects in real time on these edge-computing devices. Three scalable and fast YOLO detectors (SF-YOLO) which designed using the proposed scalable convolutional blocks compared the processing speed and accuracy with several conventional light-weight YOLO detectors on the edge-computing devices. When compared with YOLOv3-tiny, SF-YOLO was seen to be 2 times faster than the previous processing speed but with the same accuracy as YOLOv3-tiny, and also, a 48% improved processing speed than the YOLOv3-tiny-PRN which is the processing speed improvement model. Also, even in the large SF-YOLO model that focuses on the accuracy performance, it achieved a 10% faster processing speed with better accuracy of 40.4% mAP@0.5 in the MS COCO dataset than YOLOv4-tiny model.

## 1. Introduction

In an image-based object recognition technology, which is the basis of all object detection technology, the convolutional neural network (CNN) is first applied to LeNet-5 as proposed by LeCun et al. [1] in 1998. After this, CNN was then successfully applied to AlexNet as proposed by Krizhevsky et al. [2] in 2012 and it received attention after it won the ImageNet Large Scale Visual Recognition Challenge (ILSVRC) [3] with an overwhelming performance. Since after such an overwhelming performance, many researchers were approached about CNN and there were some remarkable results. A visual geometry group (VGG) networks proposed by Simonyan and Zisserman [4] had an excellent performance using a 3 × 3 size convolutional layers which had a less computational complexity than using a 5 × 5 or 7 × 7 size convolutional layers. ResNet, proposed by He et al. [5], was able to build upon the convolutional layers by applying an identity shortcut connection concept. A dense convolution network (DenseNet), proposed by Huang et al. [6], can efficiently increase the feature map channels by densely connecting (i.e., concatenating) the feature maps that have small channels.

In line with object recognition studies, there have been many studies that attempted to apply the CNN to object detection. There are two types of object detection methods that have been proposed. The first is a two-stage object detection method e.g., region-based convolution neural network (R-CNN) [7], Fast R-CNN [8], and Faster R-CNN [9]. The other is a one-stage object detection method e.g., Single Shot MultiBox Detector (SSD) [10] and You Only Look Once (YOLO) [11]. However, most of the CNN study approaches are centered around improving the accuracy performance. Despite its overwhelming accuracy performance, the CNN requires a high-performance graphic processing unit (GPU) because of its enormous amount of computational complexity. For this reason, various devices suffer from the limitations in using the CNN-based technology extensively in the real-world applications.

Object detection technology has been widely used in real-world applications long before the CNN methods became advanced. For example, face detection technology is the most basic object detection technology applied to all products relating to cameras. In 2004, Viola and Jones proposed the AdaBoost [12] method, which can detect faces in real time. This has become a breakthrough because even though the face detection technology has been studied for a long time since the 1960s, then it was difficult to apply it to products. After all, it could not process in real time up until now. The face detection technology must be performed in real time to be applied in real-world applications.

In recent years, there have been studies on light-weight CNN models such as SqueezeNet [13], MobileNet [14], ShuffleNet [15], PeleeNet [16] and MnasNet [17] for real-time processing on various edge-computing devices which have low-power consumption and low-computational performance. SqueezeNet is a light-weight model that has a very small model size with 50 times fewer parameters while having the accuracy of AlexNet level by using the 1 × 1 convolutional layers appropriately. MobileNet and ShuffleNet are light-weight models using the depthwise-separable convolution method, and the group convolution and channel shuffle methods, respectively. PeleeNet is a proposed light-weight model for mobile devices using the two-way dense layer and stem block methods, and MnasNet uses neural architecture search (NAS) to find a CNN architecture suitable for mobile devices. Furthermore, some improved CNN models were proposed by other CNN models. The improved models are MobileNetV2 [18], MoblieNetV3 [19] and ShuffleNetV2 [20].

The CNN model can achieve higher accuracy by increasing the number of convolutional layers and the number of kernels. However, the increase in the number of convolutional layers and kernels can slow down the processing speed of the CNN model. There is a trade-off relationship between the processing speed and accuracy of the CNN model. Light-weight CNN models use a smaller number of convolutional layers and kernels than the high-accuracy CNN models, which results in a high processing speed instead of decreasing the accuracy. To apply the CNN models into various edge-computing devices that have different computing powers, it is necessary to determine the processing speed and accuracy of the CNN model with regards to this trade-off relationship. However, an aforementioned light-weight CNN model has difficulty in controlling the processing speed and accuracy of the CNN model that is suitable for each edge-computing device. Furthermore, with the addition and deletion of the convolution layers, it is difficult for changes to be predicted in the processing speed and accuracy of the whole CNN model.

In terms of product development using CNN-based object detection technology, YOLO can be used to design a network model that is faster and easier than other methods. Redmon et al. released a Darknet deep learning framework with a YOLO object detector [21,22,23]. Darknet is developed with C language. It can be ported easily to various platforms and using the ’config’ file, it can easily design the CNN models. With little deep learning knowledge, developers can easily design a CNN model and use the object detection technology, even if they are not experts in deep learning. YOLO was first proposed in 2016 and constantly improved every year giving rise to YOLOv2 [21] and YOLOv3 [23] as proposed in 2017 and 2018, respectively. Along with this, the light-weight versions of YOLO such as YOLOv2-tiny and YOLOv3-tiny were also proposed [22]. Since then, improved YOLO models were proposed by other researchers such as YOLOv3-PRN, YOLOv3-tiny-PRN [24], YOLOv4 [25], and YOLOv4-tiny [26]. YOLOv3-tiny-PRN improved the processing speed by about 30% maintaining the same accuracy in NVIDIA TX2 when compared to YOLOv3-tiny. YOLOv4-tiny achieved a 40.2% mAP@0.5, which is 7.1% points higher than YOLOv3-tiny in the MS COCO dataset [27]. Nevertheless, the processing speed of YOLOv4-tiny is slightly faster than YOLOv3-tiny.

Until most recently, studies have been underway on object detection problems based on the YOLO architecture. Xu et al. [28] proposed an improved YOLOv3 using DenseNet and detected the multi-scale remote sensing targets to address the problem of poor detection performance for small objects, which is a weakness of the YOLO architecture. In light-weight YOLO for edge-computing, Wong et al. [29] proposed the YOLO Nano model by human-machine collaboration design strategy, which is 8.3 times smaller model size, 10.7% higher accuracy on VOC 2007 dataset, and 17% lower operations when compared with YOLOv3-tiny. Zhao et al. [30] proposed the Mixed YOLOv3-LITE model which combines the various CNN concepts, with achieved fast processing speed in an edge-computing device. In addition, Zhou et al. [31] performed cost evaluation on various types of hardware, such as central processing unit (CPU), graphic processing unit (GPU), application specific integrated circuits (ASIC), and field programmable gate arrays (FPGA), in order to recommend the more cost-effective hardware accelerator in various edge-computing environments. Meanwhile, FPGA can be a good approach to computing CNN networks with high efficiency. Zhang el al. [32] implemented the YOLOv2 and YOLOv2-tiny models in an FPGA and evaluated its performance.

The main contributions of this paper are as follows. First, the scalable convolutional blocks for designing scalable and fast light-weight YOLO object detectors are proposed. The scalable convolutional blocks are expected to be able to adjust the balanced processing speed and accuracy of the CNN model while considering the computing power of the various edge-computing devices. Second, the light-weight YOLO architecture named SF-YOLO, which designed by applying the proposed scalable convolutional blocks, is proposed. The SF-YOLO can be designed with the balanced processing speed and accuracy of the CNN model, noting the performance according to the computing power of the target device by exchanging the scalable convolutional blocks simply. Finally, to verify the SF-YOLO achieves faster processing speed and higher detection accuracy than the existing light-weight YOLO, the comparative experiments of the processing speed and detection accuracy in the various edge-computing devices is performed with the three state-of-the-art light-weight YOLO models.

The rest of this paper is organized as follows. Section 2 discusses the two preliminary analyses that are used to design the scalable convolutional blocks in the proposed SF-YOLO. The effects of the number of kernels of the convolutional layer on the processing speed are analyzed and the reason the processing speed of YOLOv3-tiny is slow on the edge-computing devices is found out. Section 3 describes the proposed scalable convolutional blocks and the designed novel light-weight YOLO detector using the scalable convolution blocks. In Section 4, three models of the SF-YOLO are designed. Emphasis is made on the processing speed and accuracy performance and is tested with concerns regarding the changes in the processing speed and accuracy. Comparative experiments with state-of-the-art light-weight YOLOs are discussed. In Section 5, as the ablation study, the test that changes the processing speed and accuracy with the different input dimension and the number of feature maps used in the YOLO detector is performed. The conclusions of this study are presented in Section 6.

## 2. Preliminary Analysis for SF-YOLO

In this section, the preliminary analysis to design the scalable and fast YOLO models is discussed. First, the test comparing the processing speed according to the number of kernels on the various edge-computing devices is performed. From this test, the suitable number of kernels of the convolutional layer is determined and is used to achieve the most efficient processing speed. Next, the architecture of the YOLOv3-tiny detector which is the basis of the SF-YOLO detector is analyzed. From this analysis, the reason the YOLOv3-tiny is slow in the edge-computing devices is discussed.

### 2.1. Processing Speed Comparison by the Number of Kernels

The convolutional layer consists of several kernels of size width (*w*) × height (*h*). The convolutional layer with *k* kernels transforms the width (*W*) × height (*H*) × channel (*C*) dimensions feature map into the *W* × *H* × *k* dimensions. Each of the *k* kernels can be calculated independently and simultaneously. Since the GPU can perform the computation of each kernel simultaneously through its powerful parallel processing power, it can drastically increase the processing speed of the convolutional layers.

The devices used in the edge-computing applications must be operated outdoor and exposed to various environmental conditions, unlike server-computing applications that are highly controlled indoor. Due to this exposure, the edge-computing devices have relatively low-power consumption and low-performance computing power compared to the high-performance server-computing GPU systems. The number of kernels that the edge-computing device can compute simultaneously is limited. Hence, the number of kernels of the convolutional layers has a great influence on the processing speed of the CNN models in the edge-computing devices.

**Figure 1:**
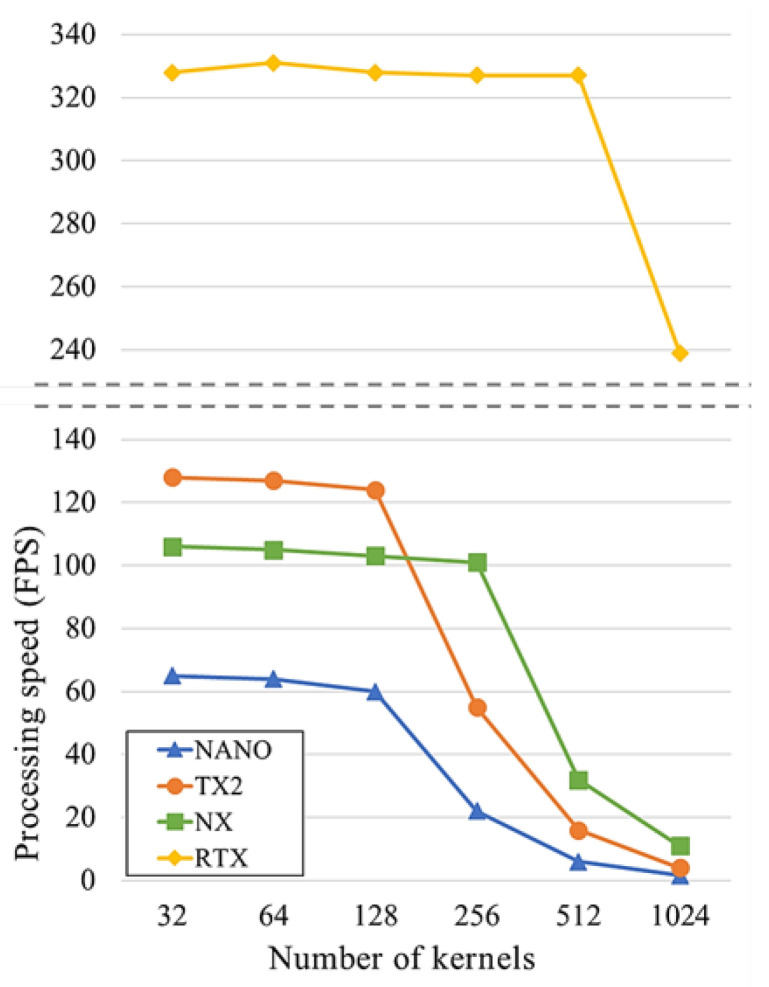
Processing speed comparison by the number of kernels on edge-computing devices.

In this subsection, we perform a test that shows the effect of the number of kernels on the processing speed by comparing experiments using three edge-computing devices. Figure 1 shows the processing speed comparison according to the number of kernels of the convolutional layers on various edge-computing devices. Jetson NANO, Jetson TX2, and Jetson Xavier NX are the GPU-based edge-computing devices released by NVIDIA. The Jetson NANO has 128-core NVIDIA Maxwell @ 921 MHz, 4 GB 64-bit LPDDR4 @ 1600 MHz | 25.6 GB/s, and up to 476 GFLOPs. The Jetson TX2 has 256-core NVIDIA Pascal @ 1300 MHz, 8 GB 128-bit LPDDR4 @ 1866 Mhz | 59.7 GB/s, and up to 1.33 TFLOPs. The Jetson Xavier NX has 384-core NVIDIA Volta @ 1100 MHz with 48 Tensor Cores, 8GB 128-bit LPDDR4 @ 1600 MHz | 51.2 GB/s, and up to 21 TOPs. Titan RTX which is one of the high-performance server-computing GPU has 4608-core NVIDIA Turing @ 1770 MHz with 576 Tensor Cores, 24 GB 384-bit GDDR6 @ 7000 MHz | 672 GB/s, and up to 16.3 TFLOPs. It has been added to the comparison list for comparison with a server-computing device.

In each device, the experiment was performed for measuring the processing speed of convolutional layers through repeating the 3 × 3 convolutional layer of *k* kernels 10 times for the color image of 32 × 32 × 3 dimensions. In each test, the kernel increased by a double from 32 until it reached 1024. In this experiment, the giga floating-point operations (GFLOPs) for the convolutional layers are calculated as follows:

(1) 2×(W×H×C)×(w×h×k)×n,

where *W*, *H*, and *C* are the width, height, and the number of channels of the feature map, *w*, *h*, and *k* are the width, height, and the number of kernels of the convolutional layer, and *n* is the number of convolutional layers of this test. Hence, if the number of kernels is 32, about 0.177 GFLOPs are required, and if it is doubled, GFLOPs increase about 2 times. The 1024 kernels require 32 times more GFLOPs than 32 kernels.

The difference in the processing speeds is significantly reduced due to the parallel processing power of the GPU. As shown in Figure 1, in NANO, the processing speed decreased slightly when the number of kernels was 128, and rapidly decreases more at 256. TX2 has no significant change in the processing speed until it reaches 128 kernels, but a significant decrease occurs at 256 and a rapid decrease occurs at 512 kernels. In the case of NX, while maintaining similar processing speed until it reaches 256 kernels, a significant decrease occurs at 512 or higher kernels. NX performs lower processing speed than TX2 in less than 128 kernels, but it achieves faster processing speed in 256 or more kernels. This is because TX2 has a single GPU clock speed of 1300 MHz, which is higher than NX’s 1100 MHz, so even though NX has higher parallel processing power, TX2 achieves faster processing speed in a small number of kernels that can perform the same parallel processing power. On the other hand, the Titan RTX which is a high-performance GPU for server-computing, maintains the same processing time even at 512 kernels, and a significant decrease occurs at 1024 kernels, but it still shows superior computing power when compared other edge-computing devices.

The experiment results show that the processing speed drops sharply at a certain number of kernels on the edge-computing devices. Therefore, it is necessary to limit the number of kernels of the convolutional layers when designing the light-weight CNN models. As seen in the results, in the currently available edge-computing devices, limiting the number of kernels to 256 or less can obtain a significant benefit in terms of the processing speed.

### 2.2. Architecture of YOLO-Tiny

**Figure 2:**
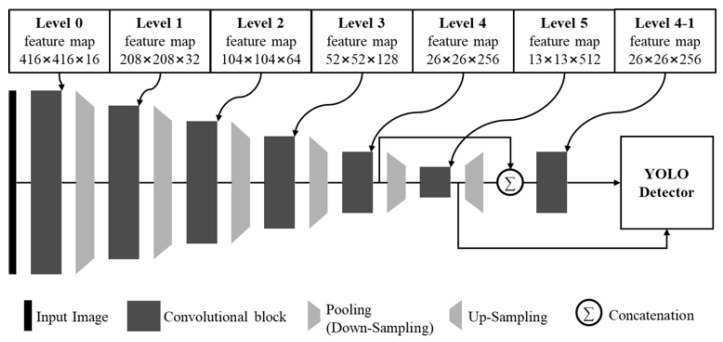
Block diagram of YOLOv3-tiny architecture.

The goal of this paper is to design a scalable and fast light-weight YOLO detector. It first starts by analyzing the state-of-the-art YOLO detector. Figure 2 shows the architecture of YOLOv3-tiny that is the light-weight version of YOLOv3. At the point of feature extraction, YOLOv3-tiny uses the five pooling layers to obtain the final feature map, and through this, the input image of *W* × *H* dimensions is converted into the final feature map of (*W*/32) × (*H*/32) dimensions. If the dimension of the input image is 416 × 416, the final feature map has 13 × 13 dimensions. However, due to the characteristics of CNN, any size is possible as long as the dimension of the input image is a multiple of 32. For example, if the input image is 320 × 320 dimensions, the dimension of the final feature map will be 10 × 10. The non-linearity of the feature map is increased by using the convolutional layers between the pooling layers. The width and height of the feature map that is passed through the pooling layer are reduced by half. After the pooling layer, the number of channels of the feature map is doubled, hence, retaining the amount of information present in the feature map. Thus, the architecture that involves doubling the number of channels while the dimension of the feature map is reduced by half. Noted that the pooling layer is used in many CNN-based feature extraction methods for studies. As described above, in the CNN-based feature extraction method, the design of the convolutional layers between the pooling layers is used to determine or affects the final feature map performance. Usually, CNN models can achieve better performance by applying more numbers of the convolutional layers. However, when the computational complexity increases exponentially, more processing time is required for feature extraction.

At the detection part, YOLO uses both the feature map of the final level and the feature map of the previous level to identify the various size objects, especially small objects by applying the concept of feature pyramid networks. YOLOv3 uses the three feature maps from level 3 to level 5, and YOLOv3-tiny uses the two feature maps, from level 4 and level 5. As the dimension of the feature map increases, the number of candidates to be considered for object detection increases rapidly, thereby increasing the processing time. The light-weight CNN model is designed to focus on the processing speed. Since it is difficult to use many convolutional layers from the lower level feature map, it is also difficult to get sufficient feature maps that were extracted at the lower level feature map. Therefore, YOLOv3-tiny uses only the two feature maps for object detection, unlike the YOLOv3.

Table 1 describes the architecture of YOLOv3-tiny when having input dimensions of 416 × 416. Until level 4, the feature maps are extracted using only one convolutional layer. In each feature map, only one convolutional layer is used to reduce the computational complexity, but enough feature maps with high accuracy cannot be gotten. Several convolutional layers and maxpool layers are used to extract enough feature maps for object detection starting from the level 5 feature map which is the final feature map. The non-linearity of the feature maps increases by using many convolutional layers. In small dimension feature maps, less computation complexity is required to extract features that were not sufficiently extracted at the previous level. Nonetheless, the convolutional layers with 512 kernels and 1024 kernels were used to extract the final feature map. According to the results of the preliminary analysis, when the kernels of the convolutional layers are 256 or higher, the processing speed decreases rapidly in the edge-computing devices.

## 3. Scalable and Fast Object Detector

In this section, while maintaining the basic structure of the YOLOv3-tiny, the scalable convolutional blocks for the SF-YOLO detector with faster processing speed and higher accuracy are implemented.

### 3.1. Scalable Convolutional Blocks for SF-YOLO

This paper aims at designing a CNN model for the scalable and fast light-weight YOLO object detector which is named as SF-YOLO. The scalable convolutional blocks are expected to be able to adjust the balanced processing speed and accuracy of the CNN model while considering the computing power of the various edge-computing devices. The proposed SF-YOLO can be designed with the balanced processing speed and accuracy of the CNN model, noting the performance according to the computing power of the target device by exchanging the scalable convolutional blocks simply.

**Figure 3:**
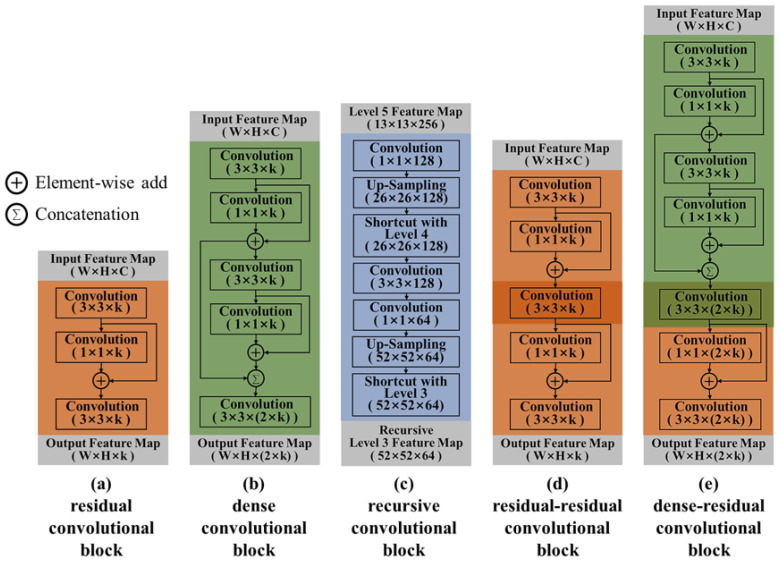
Scalable convolutional blocks.

Figure 3 shows the scalable convolutional blocks to be used in the SF-YOLO as proposed in this paper. Figure 3a is the residual convolutional block inspiring the concept of a residual network which is the basis of the SF-YOLO that is used to replace a single convolutional layer. It consists of a 3 × 3 convolutional layer and a 1 × 1 convolutional layer. The shortcut layer performs an element-wise addition of these two convolutional layers. This block analyzes the correlation between the spatial feature values of the feature map through the 3 × 3 convolutional layer and analyzes the correlation between the channels of the feature map through the 1 × 1 convolutional layer. The 1 × 1 convolutional layer was mainly used as the bottleneck layer to control the number of channels in the feature map. In this study, since the ×1 convolution layer is used to analyze the correlation between the channels in the feature map, the same number of kernels as in the 3 × 3 convolutional layer is used in this layer. The residual convolutional block affects the analysis of the neighboring feature values and the correlation between channels and results to minimize the increase in the computational complexity. Figure 3b shows the dense convolutional block concept of the dense network to double the number of channels after the pooling layer. The feature map with 2 × *k* channels is generated by concatenating the resulting feature maps output of two *k*-channel residual convolutional blocks. The dense convolutional block slightly decreases the computational complexity as compared to the method of doubling the number of channels using the single convolutional layer with 2 × *k* kernels, and the non-linearity of the feature map is increased by using the deeper convolutional layers. The recursive convolutional block in Figure 3c performs a returning function to the lower feature map while up-scaling the final feature map to compensate for insufficient feature extraction by using some little convolutional layers. The level 5 feature map of 13 × 13 dimensions is up-scaled to the feature map of 26 × 26 dimensions and an element-wise addition is performed with the level 4 feature map of 26 × 26 dimensions. All through this same process, it is recursive until it gets to the level 3 feature map of 52 × 52 dimensions. Figure 3d,e shows the scalability of the scalable convolutional blocks proposed. The residual and dense blocks can be stacked continuously. Since the scalable convolutional layers have the same size of blocks within its layers, the layers can be shared for the light-weight models. Each scalable convolutional block is expandable when necessary. While using this expandable feature, the processing speed and accuracy can be easily adjusted according to the performance of the various edge-computing devices with different computing powers.

### 3.2. Architecture of SF-YOLO

**Figure 4:**
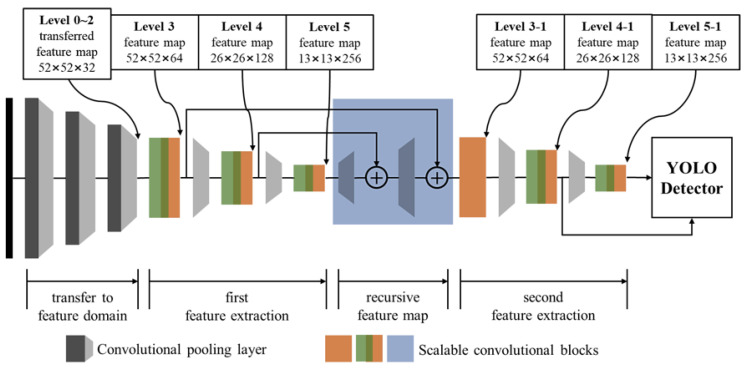
Block diagram of SF-YOLO (medium) architecture.

The SF-YOLO detector is designed by using the proposed scalable convolutional blocks. Figure 4 shows the block diagram of the proposed SF-YOLO (medium) architecture, and Table 2 describes the architecture of the SF-YOLO for cases where the input is 416 × 416 dimensions. The SF-YOLO consists of four parts namely: transfer to feature domain, first feature extraction, recursive feature map, and second feature extraction. Like the YOLOv3-tiny, the SF-YOLO transforms the input image of 416 × 416 × 3 dimensions into the feature map of 13 × 13 × *C* dimensions through the five pooling layers. From the result of the preliminary experiment, limiting the kernel of the convolutional layers to 256 or less is an advantage for the processing speed. Therefore, the number of kernels for all convolutional layers was limited to 256, hence making the number of channels in the level 5 feature map of the SF-YOLO to be 256.

YOLO detector uses the final two or three feature maps. Therefore, up to level 0 to 2 feature maps, pooling continues by using the convolutional layer with stride 2, and this part quickly transfers the input image to a feature domain. For the first feature extraction part, i.e., from level 3 to level 5 feature map, the proposed scalable convolutional blocks are used because it is necessary to increase the non-linearity of the feature maps to improve the detection performance. The maxpooling method was used for pooling from level 3 to level 5 feature map. After the pooling layer, the dense convolutional block is applied to increase the number of channels in the feature map. To increase the non-linearity of the feature maps, the dense convolutional blocks can be expanded by adding the residual convolutional blocks after the dense convolutional block such as dense-residual convolutional block in Figure 3e. Figure 4 shows an example of using the dense-residual convolutional block which extends the dense convolutional block for level 3 to level 5 feature maps and level 4-1 and level 5-1 feature maps.

It is difficult to extract enough feature maps in the light-weight CNN models with the limited kernel size and the small number of convolutional layers. To overcome this limitation, the new low-level feature map reflecting higher-level feature maps is restored with the recursive convolutional block. Since the restored recursive level 3 feature map has up to 64 channels, the level 3-1 feature map is generated using the residual convolutional block instead of the dense convolutional block. After which the level 4-1 and level 5-1 feature maps generated in the second feature extraction part are used for the YOLO detector.

**Figure 5:**
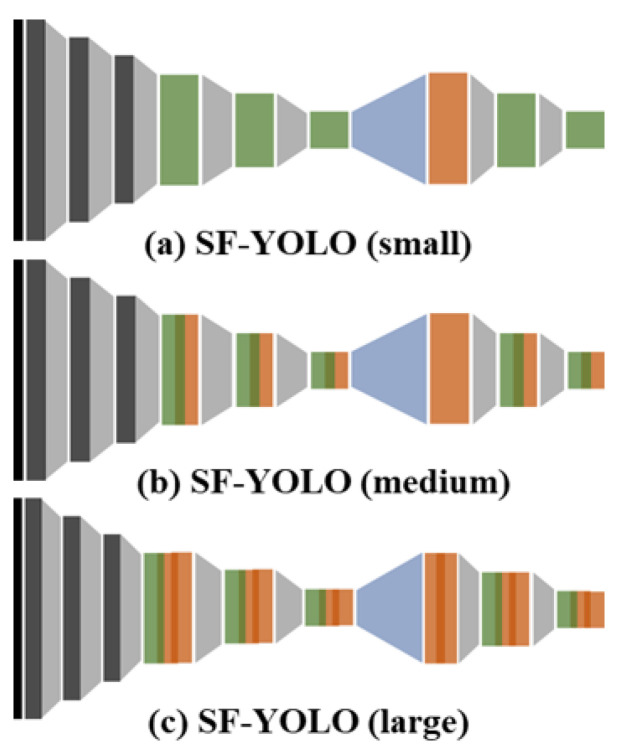
Expansion of SF-YOLO by replacing the scalable convolutional blocks.

According to the performance of the target device, the appropriately extended scalable convolutional blocks can be determined and applied to the SF-YOLO models. Figure 5 shows an example of the three SF-YOLOs which are applied to the different scalable convolutional blocks. Figure 5a shows the small SF-YOLO in its simplest form. Figure 5b shows the medium SF-YOLO in which the non-linearity of the feature map is increased as a result of the connection from the residual convolutional block to the dense convolutional block, and Figure 5c shows the large SF-YOLO that is focused on the accuracy rather than the processing speed by the addition of one residual convolutional block to each of the blocks. To design a deeper model than the large SF-YOLO model, it continuously adds to the residual convolutional blocks, but it can no longer be called the light-weight detector which is the purpose of this study. In addition to the proposed three versions of the model, there are many other extension versions, but the same scalable convolutional blocks are applied to predict the processing speed and accuracy of the models.

## 4. Experimental Results

In this section, the three proposed SF-YOLO models are tested for the processing speed and accuracy on the various edge-computing devices and are compared with the state-of-the-art light-weight YOLO detectors which are YOLOv3-tiny, YOLOv3-tiny-PRN, and YOLOv4-tiny. Excluding the architecture of the CNN model, all conditions corresponding to the hyperparameter such as the learning rate, the batch size, the number of iterates, and the number of feature maps used for detection are the same as the parameters of the YOLOv3-tiny. The training was performed in the Darknet framework, and the COCO2017 train dataset was used as training data. The accuracy was measured through the COCO evaluation server using COCO test-dev2017 dataset.

The contrast experiment for the processing speed of each of the models was performed on eight devices (four GPUs and four CPUs). First, the experiments were performed on the NVIDIA Jetson NANO, the Jetson TX2, and the Jetson Xavier NX that was released for edge-computing. The Titan RTX was used for high-performance GPU for server-computing. NANO, TX2, NX, and RTX have 128, 256, 384, and 4608 CUDA cores, respectively. Next, the experiment was also performed on Intel CPUs i.e., Core i3-8145UE, Core i5-8365UE, and Core i7-8665UE. These Intel CPUs are low power and used for embedded devices. Core i9-9900K is a high-performance CPU. Core i3-8145UE has 2 cores, 4 threads, 2.2 GHz base frequency, 3.9 GHz max turbo frequency, and 4 MB cache. Core i5-8365UE has 4 cores, 8 threads, 1.6 GHz base frequency, 4.1 GHz max turbo frequency, and 6 MB cache. Core i7-8665UE has 4 cores, 8 threads, 1.7 GHz base frequency, 4.4 GHz max turbo frequency, and 8 MB cache. Core i9-9900K has 8 cores, 16 threads, 3.6 GHz base frequency, 5.0 GHz max turbo frequency, 16 MB cache. All the 4 CPUs have the advanced vector extensions 2 (AVX2) instruction set. The 4 GPUs used the Darknet framework as their inference engine, and the 4 CPUs used the OpenCV DNN module as their inference engine. All models in this experiment used 32-bit floating-point (32FP), and OpenCV DNN inference engine supports AVX and AVX2 when running on CPUs.

Table 3 describes the results of comparing mAP@0.5 in the COCO test-dev2017 dataset and frame per second (FPS) in the 4 GPUs and 4 CPUs using the small, medium, and large SF-YOLO models and the three light-weight YOLO models. According to the results gotten from this experiment, despite the small SF-YOLO model, which is the lightest model among the SF-YOLOs, it obtained only 2.59 GFLOPs, and an mAP@0.5 of 33.1%. This is the same as YOLOv3-tiny and YOLOv3-tiny-PRN. Besides, it has a faster processing speed than the two light-weight YOLOs are applied to all GPUs and CPUs. When compared to YOLOv3-tiny and YOLOv3-tiny-PRN, the processing speed was improved by 100% and 48% in NANO, 71% and 33% in TX2, and 57% and 40% in NX, respectively. Even though NANO is the lowest-performing device, the small SF-YOLO model can be performed in real time with 34 FPS. The medium SF-YOLO model is designed to be like the GFLOPs of YOLOv3-tiny-PRN. According to the results, the mAP@0.5 was 37.7% which is 4.6% points higher than the YOLO-tiny-PRN accuracy. The processing speed was more than 10% faster on all edge-computing GPUs. Especially, the processing speed was improved by 27% in NX. The large SF-YOLO model, which focuses on the accuracy performance rather than the processing speed, was 5.02 GFLOPs, but achieved a slightly higher 40.4% mAP@0.5 as compared to the YOLOv4-tiny model which was 6.91 GFLOPs. In terms of the processing speed, the large SF-YOLO model achieved 11%, 11%, and 26% overall improvement on the YOLOv4-tiny in NANO, TX2, and NX, respectively. Compared to the YOLOv3-tiny, the accuracy was improved by 7.3% points, and the processing speed was 18%, 9%, and 18% faster, respectively. The RTX, which is a high-performance GPU, showed a very fast processing speed in the all light-weight models, and the differences between models were comparable.

Since the CPUs have a less parallel processing power than the GPUs, they cannot obtain enough processing speed as GPUs in all compared CPUs. Except for the i9 CPU, there was no significant difference in the processing speed of the devices between CPUs of i3, i5, and i7, which were all edge-computing CPUs. However, the proposed SF-YOLO models had enough processing speed for real-time processing even in the low-performance edge-computing CPUs.

Table 4 describes the comparison of GPU profile information such as GFLOPs/s, memory requirement, and power consumption for three GPU-based edge-computing devices. The YOLOv4-tiny model has the highest GFLOPs/s performance, but it needs more memory requirement and power consumption relatively. The proposed SF-YOLO models are lower in GFLOPs/s performance than the baseline models with similar GFLOPs, but they need less memory requirement and power consumption relatively.

## 5. Ablation Study

Table 5 describes the results of the additional experiments as the ablation study. Since the YOLO detector can input images of any size that are multiples of 32, the changes in the processing speed and accuracy were tested with the different sized images such as 320 × 320 and 608 × 608 which were input into the proposed SF-YOLOs. Also, the light-weight YOLO uses the final two feature maps, but the same comparison experiment was performed using the three feature maps such as the high-performance YOLOv3. There were some notable results. When the 320 × 320 images were inputs to the medium or large SF-YOLO models, a better accuracy was achieved than when the 416 × 416 images were inputs to the small or medium SF-YOLO models. There was no significant difference in the processing speed in the GPUs, but in CPUs, the small input images on the larger SF-YOLO models achieved a faster processing speed rather than the large input images on the smaller SF-YOLO models. On the other hand, when the 608 × 608 images were input to the small or medium SF-YOLO models, the accuracy improvement was not significant, although there was a decrease in the processing speed that occurred unlike the 416 × 416 images input to the medium or large SF-YOLO models. In the experiment using the three feature maps, a higher accuracy performance was achieved than using the two feature maps in all cases. There was a slight decrease in the processing speed with little significant difference in the GPUs. In the CPU, there was a 50% reduction in the processing speed. A relatively large designed model is a more efficient approach in reducing the size of the input image. If the target device is GPU-based, even if the three feature maps are used on the YOLO detector, higher accuracy can be obtained without a significant reduction in the processing speed.

## 6. Conclusions

In this paper, scalable convolutional blocks were proposed to easily adjust the balanced processing speed and accuracy in consideration of the computing power of various edge-computing devices, and also the novel scalable and fast light-weight YOLO detector was designed using these scalable convolutional blocks. To design the proposed light-weight YOLO detector, the suitable number of kernels was determined through the test on the convolutional computation speed in the various edge-computing devices, and through the analysis of the existing light-weight YOLO-tiny. The cause of the decrease in the processing speed on the edge-computing devices was analyzed. The proposed SF-YOLO was tested on the various GPUs and CPUs-based edge-computing devices, the processing speed and accuracy were compared with the state-of-the-art light-weight YOLO detectors. From the results obtained from this experiment, the proposed SF-YOLO achieved a faster processing speed and higher accuracy performance when compared to the previously existing light-weight YOLO detectors. The proposed SF-YOLO showed that it can be designed with the balanced processing speed and accuracy performance according to the computing power of the target device by exchanging the scalable convolutional blocks simply. Through the ablation study, the additional experiments were performed in more diverse conditions, and the more efficient approaches were discussed in the practical developments.

## Figures and Tables

**Table 1:** Description of YOLOv3-tiny architecture.

No.	Layer Type	Filters	Size/Stride	Output
-	input img.	-	-	416 × 416 × 3
0	conv.	16	3 × 3/1	416 × 416 × 16
1	maxpool	-	2 × 2/2	208 × 208 × 16
2	conv.	32	3 × 3/1	208 × 208 × 32
3	maxpool	-	2 × 2/2	104 × 104 × 32
4	conv.	64	3 × 3/1	104 × 104 × 64
5	maxpool	-	2 × 2/2	52 × 52 × 64
6	conv.	128	3 × 3/1	52 × 52 × 128
7	maxpool	-	2 × 2/2	26 × 26 × 128
8	conv.	256	3 × 3/1	26 × 26 × 256
9	maxpool	-	2 × 2/2	13 × 13 × 256
10	conv.	512	3 × 3/1	13 × 13 × 512
11	maxpool	-	2 × 2/1	13 × 13 × 512
12	conv.	1024	3 × 3/1	13 × 13 × 1024
13	conv.	256	1 × 1/1	13 × 13 × 256
14	conv.	512	3 × 3/1	13 × 13 × 512
15	conv.	255	1 × 1/1	13 × 13 × 255
16	YOLO	-	-	-
17	route 13	-	-	13 × 13 × 256
18	conv.	128	1 × 1/1	13 × 13 × 128
19	up-sample	-	-	26 × 26 × 128
20	route 19.8	-	-	26 × 26 × 384
21	conv.	256	3 × 3/1	26 × 26 × 256
22	conv.	255	1 × 1/1	26 × 26 × 255
23	YOLO	-	-	-

**Table 2:** Description of SF-YOLO (medium) architecture.

No.	Layer Type	Filters	Size/Stride	Output
-	input image	-	-	416 × 416 × 3
0	conv.	32	3 × 3/2	208 × 208 × 32
1	conv.	32	3 × 3/2	104 × 104 × 32
2	conv.	32	3 × 3/2	52 × 52 × 32
3–13	Dense-Res.	32 + 32	-	52 × 52 × 64
	Block	64		
14	maxpool	-	2 × 2/2	26 × 26 × 64
15–25	Dense-Res.	64 + 64	-	26 × 26 × 128
	Block	128		
26	maxpool	-	2 × 2/2	13 × 13 × 128
27–37	Dense-Res.	128 + 128	-	13 × 13 × 256
	Block	256		
38–44	Recursive	128, 64	-	52 × 52 × 64
	Block			
45–48	Residual	64	-	52 × 52 × 64
	Block			
49	maxpool	-	2 × 2/2	26 × 26 × 64
50–60	Dense-Res.	64 + 64	-	26 × 26 × 128
	Block	128		
61	maxpool	-	2 × 2/2	13 × 13 × 128
62–72	Dense-Res.	128 + 128	-	13 × 13 × 256
	Block	256		
73	conv.	255	1 × 1/1	13 × 13 × 255
74	YOLO	-	-	-
75	route 60	-	-	26 × 26 × 128
76	conv.	255	1 × 1/1	26 × 26 × 255
77	YOLO	-	-	-

**Table 3:** Processing speed comparison with state-of-the-art light-weight YOLO detectors on the edge-computing devices by COCO test-dev2017 dataset.

Model	GFLOPs	mAP@0.5	NANO	TX2	NX	RTX	i3	i5	i7	i9
		(%)	(FPS)	(FPS)	(FPS)	(FPS)	(FPS)	(FPS)	(FPS)	(FPS)
**State-of-the-art light-weight YOLOs: input dimension 416 × 416, with two feature maps (13 × 13 and 26 × 26)**										
YOLOv3-tiny	5.57	33.1	17	45	49	166	20	25	26	74
YOLOv3-tiny-prn	3.47	33.1	23	58	55	166	27	31	33	94
YOLOv4-tiny	6.91	40.2	18	44	46	165	16	22	23	64
**SF-YOLO (ours): input dimension 416 × 416, with two feature maps (13 × 13 and 26 × 26)**										
small	2.59	33.1	34	77	77	170	32	39	41	118
medium	3.69	37.7	26	62	70	168	26	33	33	96
large	5.02	40.4	20	49	58	165	21	27	28	79

**Table 4:** Comparison of GPU profile information.

Model	GFLOPs/s			Memory Requirement			Power Consumption		
				(MB)			(mW)		
	NANO	TX2	NX	NANO	TX2	NX	NANO	TX2	NX
YOLOv3-tiny	94.69	250.65	272.93	966	1044	1265	4217	6970	9517
YOLOv3-tiny-prn	79.81	201.26	190.85	921	1012	1221	3759	6537	9590
YOLOv4-tiny	124.38	304.04	317.86	1018	1053	1291	3867	7151	9696
small	88.06	199.43	199.43	899	930	1184	3520	6011	7363
medium	95.94	228.78	258.3	926	960	1210	3737	6513	8221
large	100.4	245.98	291.16	953	988	1244	3815	6825	8587

**Table 5:** Comparison between various condition of SF-YOLO.

Model	GFLOPs	mAP@0.5	NANO	TX2	NX	RTX	i3	i5	i7	i9
		(%)	(FPS)	(FPS)	(FPS)	(FPS)	(FPS)	(FPS)	(FPS)	(FPS)
**SF-YOLO (ours): input dimension 320 × 320, with two feature maps (10 × 10 and 20 × 20)**										
small	1.53	29.5	47	82	84	220	56	66	67	183
medium	2.19	33.6	35	71	84	218	43	54	55	147
large	2.97	35.8	27	54	83	217	35	44	46	121
**SF-YOLO (ours): input dimension 608 × 608, with two feature maps (19 × 19 and 38 × 38)**										
small	5.52	34.6	17	41	44	154	15	19	19	60
medium	7.89	40.6	13	31	36	153	12	16	16	48
large	10.73	43.6	10	25	30	152	10	13	13	40
**SF-YOLO (ours): input dimension 320 × 320, with three feature maps (10 × 10, 20 × 20 and 40 × 40)**										
small	1.58	31.3	43	70	84	215	24	27	27	102
medium	2.24	35.4	33	60	84	217	22	25	25	88
large	3.02	37.6	26	49	82	212	20	23	23	78
**SF-YOLO (ours): input dimension 416 × 416, with three feature maps (13 × 13, 26 × 26 and 52 × 52)**										
small	2.67	34.8	31	62	76	168	15	16	16	63
medium	3.78	39.3	24	51	65	165	13	15	15	56
large	5.11	41.3	19	42	54	164	12	14	14	47
**SF-YOLO (ours): input dimension 608 × 608, with three feature maps (19 × 19, 38 × 38 and 76 × 76)**										
small	5.71	36.0	15	31	40	152	7	8	8	30
medium	8.08	41.2	12	28	33	152	6	7	7	27
large	10.92	44.2	9	21	28	151	5	6	6	24
